# Supplementary figures and images for: Rapid generation of homogenous tumor spheroid microtissues in a scaffold-free platform for high-throughput screening of a novel combination nanomedicine
Source: PLoS One. 2023 Feb 17;18(2):e0282064. doi: 10.1371/journal.pone.0282064 (PMC9937506; doi:10.1371/journal.pone.0282064)

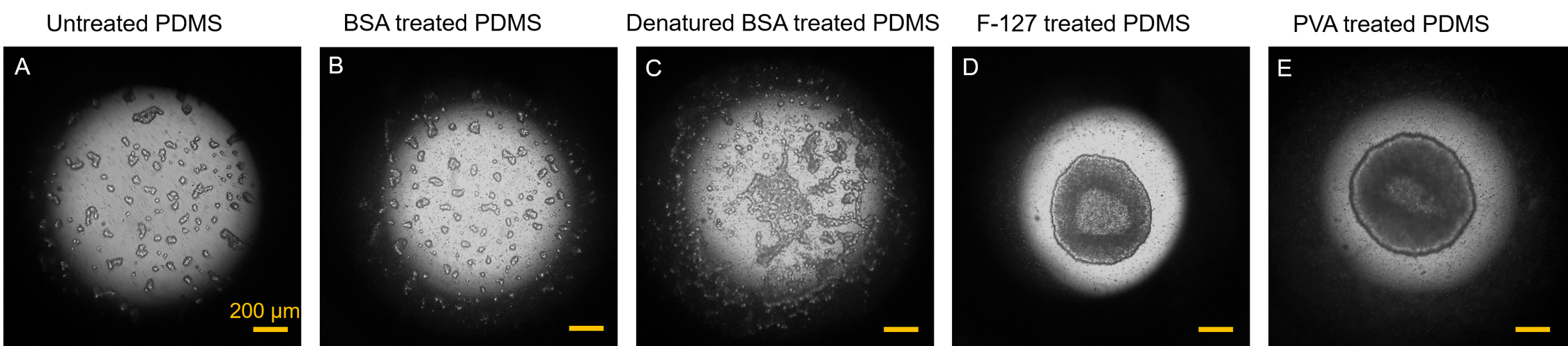

Supplement: S1 Fig — PDMS treatment with (A) Untreated, (B) 3% (w/v) BSA treated, (C) 3% (w/v) denatured BSA treated, (D) 1% (w/v) F-127 treated, (E) 1% (w/v) PVA treated. The initial cell density was 30,000 cells/well. The images were taken with 4X magnification by an optical microscope. (TIF) [file pone.0282064.s001.tif]
