# Supplementary material for: Rapid generation of homogenous tumor spheroid microtissues in a scaffold-free platform for high-throughput screening of a novel combination nanomedicine
Source: PLoS One. 2023 Feb 17;18(2):e0282064. doi: 10.1371/journal.pone.0282064 (PMC9937506; doi:10.1371/journal.pone.0282064)
Supplement: S1 Table — (DOCX) [file pone.0282064.s002.docx]

**S1 Table. Technological aspects of the presented 3D platform for tumor spheroid formation.**

| 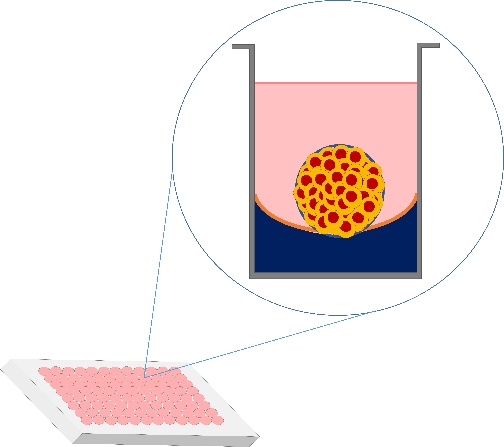 | **Spheroid culture** |
| --- | --- |
|  | High yields |
|  | High reproducibility |
|  | Uniform spheroid shape and size |
|  | Controllable size |
|  | Biorelevant tight structure |
|  | Long-term culture |
|  | Possible co-culture and hydrogel incorporation |
|  | Time-efficient |
|  | Cost-effective |
| 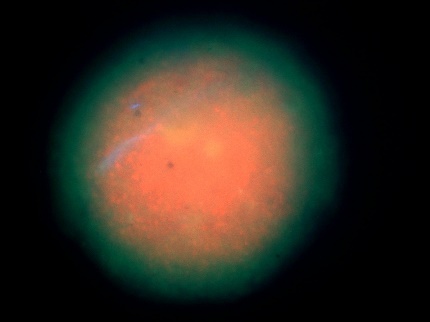 | **Drug screening and imaging** |
|  | No spheroid transfer step |
|  | Relevant physiology (signaling, oxygen, nutrient, and soluble factor gradients) and drug resistance |
|  | Compatible with high throughput screening and conventional instrumentation |
|  | Post-culture spheroid retrieval |
|  | Easy downstream processing |
