# Supplementary material for: Rapid generation of homogenous tumor spheroid microtissues in a scaffold-free platform for high-throughput screening of a novel combination nanomedicine
Source: PLoS One. 2023 Feb 17;18(2):e0282064. doi: 10.1371/journal.pone.0282064 (PMC9937506; doi:10.1371/journal.pone.0282064)
Supplement: Attachment — Submitted filename: Response to Reviewers.docx [file pone.0282064.s004.docx]

PONE-D-22-26320

Rapid Generation of Homogenous Tumor Spheroid Microtissues in a Scaffold-Free Platform for High-Throughput Screening of a Novel Combination Nanomedicine

PLOS ONE

**Response to the editor’s and reviewer’s comments and questions:**

1. Please ensure that your manuscript meets PLOS ONE's style requirements, including those for file naming. The PLOS ONE style templates can be found at https://journals.plos.org/plosone/s/file?id=wjVg/PLOSOne_formatting_sample_main_body.pdf and 
https://journals.plos.org/plosone/s/file?id=ba62/PLOSOne_formatting_sample_title_authors_affiliations.pdf

Thank you for sharing the PLOS ONE style templates. The manuscript is arranged based on PLOS ONE’s style requirements.

2. Thank you for stating in your Funding Statement: 
"This study was financially supported by Tarbiat Modares University (TMU) and Iran National Science Foundation (INSF)."

Please provide an amended statement that declares *all* the funding or sources of support (whether external or internal to your organization) received during this study, as detailed online in our guide for authors at http://journals.plos.org/plosone/s/submit-now.  Please also include the statement “There was no additional external funding received for this study.” in your updated Funding Statement. Please include your amended Funding Statement within your cover letter. We will change the online submission form on your behalf.

The below statement is added to the cover letter regarding the Funding Statement declaring all the funding or sources of support:

**Financial Disclosure**

This study was financially supported by Tarbiat Modares University (TMU) and Iran National Science Foundation (INSF). There was no additional external funding received for this study. The funders had no role in study design, data collection and analysis, decision to publish, or preparation of the manuscript.

3. In your Data Availability statement, you have not specified where the minimal data set underlying the results described in your manuscript can be found. PLOS defines a study's minimal data set as the underlying data used to reach the conclusions drawn in the manuscript and any additional data required to replicate the reported study findings in their entirety. All PLOS journals require that the minimal data set be made fully available. For more information about our data policy, please see http://journals.plos.org/plosone/s/data-availability.

Upon re-submitting your revised manuscript, please upload your study’s minimal underlying data set as either Supporting Information files or to a stable, public repository and include the relevant URLs, DOIs, or accession numbers within your revised cover letter. For a list of acceptable repositories, please see http://journals.plos.org/plosone/s/data-availability#loc-recommended-repositories. Any potentially identifying patient information must be fully anonymized.

Important: If there are ethical or legal restrictions to sharing your data publicly, please explain these restrictions in detail. Please see our guidelines for more information on what we consider unacceptable restrictions to publicly sharing data: http://journals.plos.org/plosone/s/data-availability#loc-unacceptable-data-access-restrictions. Note that it is not acceptable for the authors to be the sole named individuals responsible for ensuring data access.

We will update your Data Availability statement to reflect the information you provide in your cover letter.

As the reviewers have confirmed, all data underlying the findings described in our manuscript are fully available without restriction and the experiments have been conducted rigorously, with appropriate controls, replication, and sample sizes. The additional underlying values behind the means, standard deviations and other measures reported and the values used to build graphs in the manuscript are provided in a Supporting Information file entitled “S1 File”.

The below statement is also added to the cover letter regarding the Data Availability:

**Data Availability**

All relevant data are within the manuscript and its Supporting Information files.

4. We note that you have included the phrase “data not shown” in your manuscript. Unfortunately, this does not meet our data sharing requirements. PLOS does not permit references to inaccessible data. We require that authors provide all relevant data within the paper, Supporting Information files, or in an acceptable, public repository. Please add a citation to support this phrase or upload the data that corresponds with these findings to a stable repository (such as Figshare or Dryad) and provide and URLs, DOIs, or accession numbers that may be used to access these data. Or, if the data are not a core part of the research being presented in your study, we ask that you remove the phrase that refers to these data.

Citations are added to the statement as follows:
“The entire content of free drugs can be released within hours (11, 27).” (Section 3.4. In vitro release, page 16)

**5. Review Comments to the Author**

Reviewer #1: The authors presented a study on the screening of nanomedicine on tumor spheroids.
They constructed the adhesive-free concave surface and loaded the assembled nanodrugs for testing tumor inhibition efficiencies at varied component ratios and delivery methods. They addressed on the surface treatment, drug synthesis and drug intake quantitation process and tested the drug efficacy in 3D spheroids. However, issues listed below should be settled before being accepted.

Thank you very much for your valuable time and for sharing your helpful comments.

1. Page 16, line 1. Typos in “MDA-MB-23”. The missing number should be implemented.

The manuscript was double-checked and errors were corrected. The changes made to the manuscript were highlighted in the “'Revised Manuscript with Track Changes” file.

2. The figures should be combined together to include more information in one figure. And, the quality (font consistency, color matching, boldness, arrows, resolution) should all be improved profoundly. There must be neat ways of exhibition for Figure 3, 4, 12.

The figures are comprehensively edited and combined. The figures are also edited and standardized using the PACE service of PLOS ONE as well.

**Figure 1. 3D platform for spheroid culture.** (A) Spheroid culture process onto the non-adherent concave bed in a 96-well plate. (B) peeled off PDMS layer from a well.

**Figure 2.** **5FU-CUR-PIP-HSA-NPs characterization.** (A) SEM image and (B) particle size distribution of 5FU-CUR-PIP-HSA-NPs. (C) DSC thermograms of 5FU, PIP, CUR, HSA, and 5FU-CUR-PIP-HSA-NPs. (D) In vitro release patterns of the three drugs from 5FU-CUR-PIP-HSA-NPs in different buffers at 37 °C. Each point represents the average of three independent experiments, and error bars show the SD.

**Figure 3.** **Formation of tumor spheroids.** (A) Sequential images of spheroid culture with the initial cell density of 30,000 cells/well. The images were taken with 4X magnification by an optical microscope. Scale bars = 200 μm. (B) SEM images of the generated MDA-MB-231spheroid with the initial density of 40,000 cells per spheroid on day 7 with different magnifications.

**Figure 4. Tumor spheroid characterization.** (A) Brightfield image of the tumor spheroid and the same image with pathophysiological concentric zones. Scale bar = 100 μm (B) Fluorescent image of live/dead assay on a tumor spheroid using FDA (green) and PI (red)). Scale bar = 200 μm. (C) Flow cytometric annexin V/PI analysis of MDA-MB-231 spheroids after 4 days. Quadrant assignment: lower left = viable cells; lower right = early apoptotic cells; upper right = late apoptotic cells; upper left = necrotic cells.

**Figure 5. Penetration of therapeutics/nanomedicine (5FU-CUR-PIP-HSA-NPs) in MDA-MB-231 cancer cells in 2D and 3D conditions.** In vitro uptake of (A) free drugs combination and (B) 5FU-CUR-PIP-HSA-NPs in 2D monolayer cancer cells at the CUR/5FU/PIP concentration of 50/26.8/7.4 μg/ml after 4 h. Scale bars = 50 μm. In vitro uptake of (C) free drugs combination and (D) 5FU-CUR-PIP-HSA-NPs with different concentrations and incubation times in 3D tumor spheroids. The green fluorescence represents CUR. Scale bars = 200 μm.

**Figure 6. Cytotoxicity and anti-metastatic activity of 5FU-CUR-PIP-HSA-NPs on MDA-MB-231 cancer cells.** (A) Anticancer activity of free drugs combination and 5FU-CUR-PIP-HSA-NPs on MDA-MB-231 monolayer (2D) and spheroids (3D) after the 48 h treatment. Each represented data point is calculated as the mean ±SD of four experiments. (B) Migration inhibition of metastatic MDA-MB-231 cells by wound healing assay. After the scratch creation, cells were treated with 5FU-CUR-PIP-HSA-NPs. After 24 h, the wound’s gap became completely covered in the control group, meanwhile, the migration of metastatic cells was suspended using a low concentration of the NPs. Scale bars = 50 μm.

3. Some of the figures are supposed to move into supp. Figures.

The figure of PDMS surface treatment and Table 1 with its figures are entitled “S1 Figure” and “S1 Table”, respectively, and moved to the Supporting Information files.

**S1 Figure.** **Surface treatment for spheroid culture.** PDMS treatment with (A) Untreated, (B) 3% (w/v) BSA treated, (C) 3% (w/v) denatured BSA treated, (D) 1% (w/v) F-127 treated, (E) 1% (w/v) PVA treated. The initial cell density was 30,000 cells/well. The images were taken with 4X magnification by an optical microscope.

**S1 Table. Technological aspects of the presented 3D platform for tumor spheroid formation.**

|  | **Spheroid culture** |
| --- | --- |
|  | High yields |
|  | High reproducibility |
|  | Uniform spheroid shape and size |
|  | Controllable size |
|  | Biorelevant tight structure |
|  | Long-term culture |
|  | Possible co-culture and hydrogel incorporation |
|  | Time-efficient |
|  | Cost-effective |
|  | **Drug screening and imaging** |
|  | No spheroid transfer step |
|  | Relevant physiology (signaling, oxygen, nutrient, and soluble factor gradients) and drug resistance |
|  | Compatible with high throughput screening and conventional instrumentation |
|  | Post-culture spheroid retrieval |
|  | Easy downstream processing |

Reviewer #2: In this work, the authors show how three compounds encapsulated in nanoparticles are used to test drug efficacy in 2D cell cultures and 3D spheroids obtained from a breast cancer cell line.
Overall, the paper lacks novelty and clarity in few aspects of the proposed work. Therefore, I do not recommend publication in its present form. Below are my comments:

Thank you very much for your time and for sharing your constructive comments on our manuscript.

1) The introduction is rather lengthy and it would benefit from a clear summarised statement with regard to what aspect(s) of in vitro preclinical assays this work is addressing.

The introduction part is edited and summarized and the original in vitro preclinical aspects of the work are addressed with more clarity.

2) It is unclear what the original aspects of this work are. A statement of novelty should be inserted. Is this about the NP formulation? Running NP-based assays in low adhesion plates using spheroids is not novel, so maybe is the results obtained? These points should be clearly addressed to remove ambiguities.

A detailed description of the original aspects of our work and its novelties are provided below to remove ambiguities. The novel aspects of the work are highlighted in different parts of the manuscript (abstract, introduction, results and discussion, and conclusion).

Novel aspects of our work include both the synthesis of a triple drug delivery system and the establishment of a versatile in-house developed platform in 96-well plate for spheroid culture. Exploiting different mechanisms of action, a novel triple drug delivery system of 5-fluorouracil, curcumin, and piperine co-loaded human serum albumin nanoparticles (5FU-CUR-PIP-HSA-NPs) was developed via the self‐assembly method for suppressing breast tumor tissue. The drug delivery system has the ability to encapsulate both hydrophobic and hydrophilic drugs simultaneously in a single carrier with a high drug loading efficiency. Furthermore, the drug delivery system can serve as both therapeutic and diagnostics with proper physical properties and benefit passive targeting through the EPR effect. On the other hand, employing the liquid overlay technique principles, a promising scaffold-free platform was developed in standard 96-well flat plates in a cost-effective novel approach for rapid generation of homogenous tight 3D tumor spheroid microtissues and anticancer therapeutic/nanomedicine screening. The technological aspects and superiority of the developed platform over other commonly used spheroid culture techniques like liquid overlay (by agar/agarose coating), hanging drop, magnetic levitation, micropatterned plates, spinner flasks, matrix encapsulation, and scaffolds/hydrogels were discussed in the manuscript and summarized in S1 Table.

The existing challenges in spheroid culture are elaborated and addressed by providing statements regarding our proposed platform. One frequently used method for spheroid generation is the liquid overlay method [1]. In the ultra-low attachment plates, generally, the main substrates used to coat the plate are agar/agarose or poly(2-hydroxyethyl methacrylate), by adding 50 μL of solution in each well of 96-well plates, at concentrations of 10-15 mg/mL and 5mg/mL, respectively [2]. Agar/agarose [3-10] and poly(2-hydroxyethyl methacrylate) [11, 12] coated 96-well plates and commercially available low adhesion 96-well plates [13-16] have been extensively used for spheroid culture and drug screening.

However, the variability of the size and/or spherical morphology of the spheroids, low efficiency, limited visualization, lack of reproducibility, and difficulty in long-term culture have been counted as disadvantages for most employed ultra-low attachment plates [17-22]. The biomaterials used for the non-adherent surface culture so far, could have interactions with the drug or nano-carriers, thus affecting the accuracy of the results acquired from spheroid models as well [23].

The short storage time of the coated plates has been considered the main disadvantage of using agar/agarose in the liquid overlay technique [18]. Water evaporation and consequently the coating disruption can occur in such covered plates during various days and result in impairing the reproducible production of the spheroids. Furthermore, long-term culture may be needed for spheroids to form and become dense for biological activity tests [4, 6, 7, 24]. transferring step for subsequent drug treatment, imaging, and analysis is majorly required in such coated plates [3] increasing the risk of disaggregation and/or loss of spheroid structure.

Likewise, the utilization of poly(2-hydroxyethyl methacrylate), as a non-adherent material for the production of spheroids through the liquid overlay technique, is relatively expensive and laborious [18]. Moreover, compared with in-house coated plates, commercially available low-adhesion 96-well plates are relatively expensive [25-27].

PDMS, as a biocompatible cost-effective polymer with several excellent material properties for biomedical applications provides a chemically inert surface with low interfacial free energy, good thermal stability, and optical transparency. The surface properties of PDMS are also relatively easy to modify [28]. In our work, by using PDMS casting in 96-well plates, an in-house U-shape design to the bottom of the culture plate wells was established (concave bottom) and modified with PVA to prevent cell adhesion to the surface and facilitate single and well-defined round-shaped spheroid formation per wells.

**Figure. 3D platform for spheroid culture.** (A) Spheroid culture process onto the non-adherent concave bed in a 96-well plate. (B) peeled off PDMS layer from a well.

when using flat non-adherent surfaces with a thin layer of non-adherent biomaterial, several irregular cellular aggregates with non-spherical shapes have been obtained by the liquid overlay technique [18]. These cellular aggregates do not meet the requirements to be used in high-throughput screening, which hinders the translation of these technologies to the industry [18]. The round-shaped single spheroids cultured in our model can benefit mathematical modeling too [18]. With the calculated diameter in the round-shaped tumor spheroids produced in our platform, a good estimate can be performed on the volume ($V=\frac{1}{6}\left( \pi D^{3} \right)$) and the number of cells within spheroids and drug/nanodrug distribution across the spheroids can be mathematically modeled.

In addition, due to the difficulty to obtain homogenous spheroid cultures, a high throughput screening on spheroids is limited [3]. Scaffold-free systems in 96-well plates format are currently the most amenable to high-throughput screening, due to their compatibility with conventional liquid handling and robotic equipment [29]. In this regard, large number of homogenous highly reproducible spheroids were produced in the prepared platform, compatible with the high-throughput screening assays. Using this 3D culture platform, spheroids can be arrayed and utilized to screen chemical or RNAi/CRISPR libraries in combination with a variety of assay readouts such as size, viability, and imaging without transferring steps. The effect of the chemical compounds like therapeutics on a variety of parameters such as viability, MTS size, reporter genes, or fluorescent dye intensity can also be assessed [29]. Accordingly, the use of spheroids can be extended for more practical disease modeling and high-throughput screening.

Altogether, combining two major approaches of disease modeling through the spheroid formation and drug delivery, remarkable results were obtained. The explanations and points are added to the manuscript to justify it and the aims of the work are discussed accordingly. The added explanations to the manuscript are as follows:

“The aim of this study was to develop a homemade preclinical 3D culture model in the standard 96-well plates in a cost and time-effective novel approach for the rapid generation of homogenous compact tumor spheroids for disease modeling, and anticancer therapeutic/nanomedicine screening.” (Abstract, Page 2)

“Using such a delivery system, both hydrophobic and hydrophilic drugs can be simultaneously encapsulated with a high drug loading efficiency (DLE).” (Section 1. Introduction, page 4)

“To address these, herein, we established a preclinical scaffold-free 3D spheroid culture model in the standard 96-well flat plates by employing a homemade set-up. The novel aspect of our 3D platform includes the generation of a large quantity of uniform tight single spheroids in a cost and time-effective approach for drug screening with no necessary transfer step. Using this new in situ high throughput compatible 3D model system alongside 2D culture platforms, we investigated the efficacy of the formulated drug delivery system (5FU-CUR-PIP-HSA-NPs) to triple negative MDA-MB-231 breast cancer cells. The study provides proof-of-concept results for employing the 3D culture system to test the biological activities of the drug delivery system.” (Section 1. Introduction, page 5)

“To exploit different mechanisms of action in breast tumor treatment, the triple drug delivery system of 5FU, CUR, and PIP was synthesized using the self-assembly method. Both hydrophobic and hydrophilic drugs were successfully encapsulated in the HSA NPs as a single drug delivery system.” (Section 3.1. 5FU-CUR-PIP-HSA-NPs preparation and characterization, page 14)

“In the ultra-low attachment plates, agar/agarose and poly(2-hydroxyethyl methacrylate) (pHEMA) are mainly used to coat the plates for spheroid culture (4). The transferring step need for subsequent bioassays and the short storage time of the coated plates have been considered the main disadvantage of using agar/agarose in the liquid overlay technique (30, 31), while long-term culture may be needed for spheroids to form and become dense (51, 52). In addition, when using flat non adherent surfaces, several irregular cellular aggregates can be formed limiting the high-throughput screening applications (30). Besides, pHEMA is relatively expensive, and preparing the pHEMA-coated plates is laborious (30). Moreover, compared with homemade coated plates, commercially available low-adhesion 96-well plates are relatively expensive (53).” (Section 3.5. Formation and characterization of multicellular tumor spheroids, page 18)

“To address these challenges, a particular treated concave-based bed in a 96-well plate (high throughput compatible) with a U-shape design was established in this study for rapid one spheroid formation in each well. PDMS, as a biocompatible cost-effective polymer with several excellent material properties for biomedical applications provides a chemically inert surface with low interfacial free energy, good thermal stability, and optical transparency. The surface properties of PDMS are also relatively easy to modify (54).” (Section 3.5. Formation and characterization of multicellular tumor spheroids, page 18)

“Spheroid cultures of uniform sizes and growth characteristics are critical factors that considerably affect the accurate quantification of biological or biochemical endpoints in drug/nanomedicine screening (31).” (Section 3.5. Formation and characterization of multicellular tumor spheroids, page 19)

“Compared with the conventional techniques like hanging drop, this platform can rapidly generate reproducible homogenous large tumor microtissues with high compactness paving the way for a variety of high throughput screening assays and readouts with no transferring steps.” (Section 3.5. Formation and characterization of multicellular tumor spheroids, page 19)

“Such round-shaped spheroids produced in this model can benefit mathematical modeling too (30).” (Section 3.5. Formation and characterization of multicellular tumor spheroids, page 20)

“As a practical 3D model, a novel scalable scaffold-free production of controllable tumor spheroids platform was established in this study by utilizing a homemade set-up for disease modeling and anticancer drug/nanomedicine screening” (Section 4. Conclusion, page 26)

3) The authors state the importance of using physiologically relevant models of cancer to bridge the gap with in vivo models, but here a single cell line is used which is far from any TME complex composition. So one may question the suitability of the model used (for example due to the lack of ECM, fibroblasts or other cell types in an avascular tumour tissue).

3D bioengineered tumor models (e.g. spheroids, organoids, bioprinting, and organ-on-a-chip) are emerging to bridge the gap between 2D culture platforms and *in vivo* animal models [30, 31]. Multicellular tumor spheroid models (MCTS) are reported to be suitable models to gain additional information on nanoparticles. While not being able to replace in vivo studies, they can bridge the gap between traditional 2D in vitro studies and in vivo models [1]. Our tumor spheroid model can massively generate spheroids with low cost and short duration while rendering various characteristics of the in vivo avascular tumor tissue and can enhance drug screening in a high throughput manner. Large tumor spheroids (>500 μm in diameter as produced in this study) are capable of mimicking various properties of human solid tumors with sizes from 0.5 to 1 mm^3^ [32]. They resemble to *in vivo* avascular tumor tissue conditions in terms of shape and cellular architecture, tightness, cell–cell and cell-ECM physical interactions, proliferation gradients, protein and gene expression patterns, hypoxia, acidic microenvironment, physical barriers, and drug penetration and sensitivity [17, 32, 33]. Due to mass transport limitations and a 3-D structure similar to in vivo tissues, drug penetration limitations occurring in vivo can be studied in spheroids [34]. Hence, the model we have developed mirrors at least some characteristics of *in vivo* avascular tumor tissues and can serve as an intermediate model between monolayer cultures and in vivo models in high throughput screenings; while the platform can be boosted by integrating other TME components like ECM and stromal cells into it.

It is worth noting that, the tumor cells, themselves, are reported to be able to express high levels of various ECM molecules like fibrillar collagens, fibronectin, elastin, and laminins in solid tumors [35, 36]. MDA-MB-231 cells can produce ECM molecules like tenascin-C, laminin, laminin, and fibronectin [37]. Although single cell type was used for spheroid formation and screening in this study, the practicability of the platform was tested for different cell cultures and co-culture. The generation of MCF-7 tumor spheroids and co-culture spheroids (MDA-MB-231/MCF-7 cells and fibroblasts) was feasible using this platform. As a result, employing this practical 3D platform, can pave the way for recapitulating more advanced models for disease modeling and drug screening in advance.

In fact, the platform has the potential to replicate TME with more complexity by incorporating other TME compositions like ECM biomaterials and stromal cells. By integrating other elements, our high throughput compatible platform can enhance the translational predictive value of *in vitro* drug evaluation studies and provide a reduction in both financial and time costs during later stages of the drug development timeline, allowing the early detection of ineffective agents, thus reducing the risk of drug withdrawal from the market [30]. The procedure has the potential to be extended to primary patient samples (organoids) for drug screening as well.

The below explanations are added to the manuscript to address the point as well :

“The generation of MCF-7 tumor spheroids and co-culture spheroids (MDA-MB-231/MCF-7 cells and fibroblasts) was also feasible using this platform.” (Section 3.5. Formation and characterization of multicellular tumor spheroids, page 19)

“Spheroid cultures of uniform sizes and growth characteristics are critical factors that considerably affect the accurate quantification of biological or biochemical endpoints in drug/nanomedicine screening (31). Compared with the conventional techniques like hanging drop, this platform can rapidly generate reproducible homogenous large tumor microtissues with high compactness paving the way for a variety of high throughput screening assays and readouts with no transferring steps.” (Section 3.5. Formation and characterization of multicellular tumor spheroids, page 19)

“The developed 3D platform mirrors at least some characteristics of the in vivo avascular tumor tissues and can serve as an intermediate step between monolayer cultures and in vivo models while the platform can be boosted by integrating other tumor microenvironment components like ECM and stromal cells into it.” (Section 4. Conclusion, page 26)

“Translation of the employed 3D platform to wider applications (co-culture spheroids and organoid generation) for advanced drug screening is in development.” (Section 4. Conclusion, page 27)

4) The bottom image in the table on page 10 is blurry and it is unclear what is representing. The table itself is redundant and the points have already being discussed in the introduction.

It is the fluorescent image of live/dead assay on the tumor spheroid using FDA (green) and PI (red)). The figure was brought into the table to serve as a representative of biological assays which are feasible in this platform and now is replaced with a better live/dead figure. The table is moved to the Supporting Information files as “S1 Table” as well.

5) Very importantly, what is the advantage of using the proposed NPs if the drug efficiency was higher for free drugs (i.e. without NPs)? What is the evidence of “enhanced penetration and combinational therapy effect were practically translated into the tumour spheroid cytotoxicity after a 48 h treatment” as stated in section 3.7? I cannot find this in the paper.

Align with the previously published reports [38-43], higher anticancer activity can be observed for free drugs compared with their nanoformulations both *in vitro* and *in vivo* as the drugs need time and driving force to be released inside the cells. Similar results to this work were observed in the study by Kim et al [39]. In fact, the slow and partial sustained release of cargoes can be responsible for this phenomenon. This delay of action was reflected in their anticancer activities. A delayed cytotoxic response in 2D monolayer, spheroids, and SiHa xenograft mice *in vivo* was observed with doxorubicin-loaded micelle compared with free doxorubicin possibly resulting from the controlled drug release profile [39]. Free drugs administration is mainly limited by poor water solubility, non-specificity, systemic toxicity, and low plasma half-life. Compared to its nanoformulation, free doxorubicin showed excellent tumor reduction but with significant systemic toxicity *in vivo*. On the other hand, longer blood circulation was observed for doxorubicin-micelle compared to free DOX [39].

In intravenous administration, when a combination of free drugs are administered systematically, they undergo different mechanisms of metabolism and elimination, resulting in diverse plasma half-lives, biodistributions, and the final drug concentrations reaching the tumor sites which can hamper the clinical efficacy [44, 45]. The efficacy of the drugs used in our study is limited in free native form due to poor water solubility, instability, non-specificity, systemic toxicity, low plasma half-life, and MDR effect [46-48] A meticulous design of the nano-drug delivery systems is one of the solutions to address these challenges [45]. Nanoparticles are capable of delivering anticancer drugs to tumor sites adequately with higher stability and bioavailability while decreasing their side effects [49, 50]. In this context, the co-encapsulation approach displays the advantage of synchronizing the pharmacokinetics of different agents, co-localizing the three drugs into the same tumor site, and maintaining a predetermined synergistic drug ratio, results in an enhanced efficacy [45]. The synthesized drug delivery system in this study (5FU-CUR-PIP-HSA-NPs) can be a promising drug delivery system to deliver both hydrophobic and hydrophilic drugs in a single biocompatible nanosystem with different mechanisms of action, decrease the systemic distribution of free drugs, and enhance the drug accumulation in the tumor via the enhanced permeability and retention (EPR). Furthermore, they have the potential to be grafted by targeted ligands to enhance tumor targeting.

The below statements are added to the manuscript regarding the advantages of using NPs over free drugs:

“By encapsulating all these three drugs in a highly biocompatible nanocarrier i.e. HSA-NPs with proper physiochemical properties, the limitations associated with administration of the free drugs like poor water solubility, non-specificity, systemic toxicity, low plasma half-life, and MDR effect can be overcome and a tremendous effect of cancer theranostics can be achieved (5, 11, 15). Using such a delivery system, both hydrophobic and hydrophilic drugs can be simultaneously encapsulated with a high drug loading efficiency (DLE).” (Section 1. Introduction, page 4)

“To exploit different mechanisms of action in breast tumor treatment, the triple drug delivery system of 5FU, CUR, and PIP was synthesized using the self-assembly method.” (Section 3.1. 5FU-CUR-PIP-HSA-NPs preparation and characterization, page 14)

“Both hydrophobic and hydrophilic drugs were successfully encapsulated in the HSA NPs as a single drug delivery system.” ((Section 3.1. 5FU-CUR-PIP-HSA-NPs preparation and characterization, page 14)

“Similar results were observed in the study by Kim et al. (66).” (Section 3.7. Anticancer activity in 2D and 3D culture platforms, page 23)

“The co-delivery approach provides the advantage of synchronizing the pharmacokinetics of different therapeutics, co-localizing the three drugs into the same tumor region, and maintaining a predetermined synergistic drug ratio leading to an enhanced efficacy (67).” (Section 3.7. Anticancer activity in 2D and 3D culture platforms, page 24)

Regarding the second part of the question, the cytotoxicity outcome from the MTT assay showed remarkable results in suppressing triple-negative breast cancer for the NPs. At the CUR/5FU/PIP concentration of 50/26.8/7.4 μg/ml, the NPs passed the half maximal inhibitory concentration (IC_50_) in both 2D and 3D culture platforms. In fact, enhanced penetration and combinational therapy effect previously observed in *in vitro* uptake test were practically translated into the tumor spheroid cytotoxicity after the 48 h treatment and NPs induced cancer cell suppression. At the CUR/5FU/PIP concentration of 50/26.8/7.4, a widespread homogenous radial distribution was previously observed for the NPs across the tumor spheroids including the necrotic core region demonstrating the practicality of the nanomedicine system. Proper size, zeta potential, and drug combination of the prepared drug delivery system in albumin NPs could be responsible for the facilitated effective deep penetration into the solid tumors and their anticancer activity.

The statement is more clarified as follows:

“The enhanced penetration and combinational therapy effect, previously observed in the in vitro uptake test were practically translated into the tumor spheroid cytotoxicity after the 48 h treatment (Fig 6A).” (Section 3.7. Anticancer activity in 2D and 3D culture platforms, page 24)

6) It is well established that 2D cell monolayer respond differently to 3D cultures, but the model used here is not similar to any in vivo tumours.

Several 3D tumor culture models, including tumor tissue explant models, scaffold-based models, microfluidic models, and multicellular tumor spheroids have been reported to bridge the gap between *in vitro* and *in vivo* [30]. Among them, spheroid models attract extensive attention because they well preserve the 3D structure as well as the penetration barriers of the tumor tissue created for therapeutics. Spheroid models can also be massively generated with low cost and short duration. Recently, spheroid models have been employed in a number of studies for the evaluation of drug or gene delivery systems [23]. Spheroids with a diameter larger than 400–500 μm retain the characteristics of tumors in human bodies well with protein and gene expression profiles and create substantial barriers for drugs to penetrate. They can sustain for 2 weeks and thus are available for long-duration research. In addition, it is relatively convenient to carry out mathematical modeling and subsequent *in vitro* tests on spheroid models, such as cell viability analysis and imaging [23].

Compared to 2D culture platforms, large tumor spheroids (>500 μm in diameter) are capable of mimicking various properties of human solid tumors with sizes from 0.5 to 1 mm^3^ [32]. They resemble to *in vivo* avascular tumor tissue conditions in terms of shape and cellular architecture, tightness, cell–cell and cell-ECM physical interactions, proliferation gradients, protein and gene expression patterns, hypoxia, acidic microenvironment, physical barriers, and drug penetration and sensitivity [17, 32, 33]. Due to mass transport limitations and a 3D structure similar to in vivo tissues, drug penetration limitations occurring in vivo can be studied in spheroids [34].

Spheroid models have been widely used as an intermediate step in anti-tumor drug delivery system studies and have been shown to provide authentic predictions for their efficacy *in vivo* [2, 23]. Despite some limitations, the evaluation of formulation efficacy in spheroids rather than monolayer cultures is expected to more accurately reflect therapeutic performance *in vivo* [15]. While using scaffold-free homotypic cells, good correlations between *in vitro* tumor spheroids and *in vivo* animal tests were reported in several studies for nanomedicines assessment [6, 10, 22, 24, 43, 51-53] demonstrating the potential and practicality of tumor spheroids (similar to the spheroids produced in our study) in nanomedicine screening and predicting in vivo efficacy.

In this study, large tumor spheroids were produced in a time and cost-effective manner. The model we have developed mirrors at least some characteristics of *in vivo* avascular tumor tissues and can serve as an intermediate model between monolayer cultures and in vivo models in high throughput screenings; while it can be boosted by incorporating other TME components like ECM and stromal cells. It is reported that spheroids in the near future will be imposed as the gold standard *in vitro* model demonstrating a high translational predictive value, reducing the number of *in vivo* studies to be conducted as well as expediting the drug discovery process [32, 34].

The below statements are added to the manuscript regarding the biorelevancy of produced tumor spheroids with the potential high throughput screening:

“Spheroid cultures of uniform sizes and growth characteristics are critical factors that considerably affect the accurate quantification of biological or biochemical endpoints in drug/nanomedicine screening (31). Compared with the conventional techniques like hanging drop, this platform can rapidly generate reproducible homogenous large tumor microtissues with high compactness paving the way for a variety of high throughput screening assays and readouts with no transferring steps.” (Section 3.5. Formation and characterization of multicellular tumor spheroids, page 19 )

“The developed 3D platform mirrors at least some characteristics of the in vivo avascular tumor tissues and can serve as an intermediate step between monolayer cultures and in vivo models while the platform can be boosted by integrating other tumor microenvironment components like ECM and stromal cells into it.” (Section 4. Conclusion, page 26)

7) Regarding the wound healing assays, why was this not done with free drugs as well as a comparison? The conclusion obtained from this assay are not meaningful in my opinion without a comparison with free drugs.

The NPs formulation after demonstrating a great potential in biological activities (in vitro uptake and tumor cytotoxicity were further assessed for anti-metastatic activity. As a final test, we evaluated the cell migration activity treated with the proposed drug delivery system. Indeed, the free drugs combination was also used for the wound healing assay. Subtoxic concentrations are normally used for the wound healing assay to minimize the cell-killing effect of the formulations [54]. According to the anticancer activity test results (MTT assay), the CUR/5FU/PIP concentration of 10/5.4/1.5 used for this study is lower than the IC_50_ of the NPs (CUR/5FU/PIP concentration of 20.6/11.1/3 μg/ml) and had a low cell-killing effect on MDA-MB-231 cancer cells. On the other hand, the CUR/5FU/PIP concentration of 10/5.4/1.5 is near the IC_50_ value for free drugs combination. The inhibitory effect of free drugs was considerably greater than the NPs formulation at that concentration. Therefore, as we expected, after 24 h exposure, cells seemed to be killed (Figure below).

**Figure.** Migration inhibition of metastatic MDA-MB-231 cells by wound healing assay. After the scratch creation, cells were treated with 5FU-CUR-PIP-HSA-NPs and free drugs combination.

In fact, since at low concentrations, there are significant differences between the anticancer activities of the free drugs combination and the NPs formulation, the free drugs combination group cannot provide a reliable comparison for the NPs formulation. As a result, the migration inhibitory effect of the NPs was compared with the untreated cells (control group) as performed in some studies [55-60].

The below statement is added to the manuscript in this regard:

“A subtoxic concentration (CUR/5FU/PIP concentration of 10/5.4/1.5 μg/ml), lower than the IC_50_ of the NPs (CUR/5FU/PIP concentration of 20.6/11.1/3 μg/ml), was used to minimize its cell-killing effect (37). As shown in Fig 6A at low concentrations, there are significant differences between the anticancer activities of the free drugs combination and the NPs formulation. Consequently, since the free drugs combination group causes a high cell-killing effect, it cannot provide a reliable comparison for the NPs formulation in the wound healing assay.” (Section 3.8. Migration-cell wound closure assay, page 26)

8) Where is the evidence of deep NP penetration? It is stated that NPs could not penetrate tight spheroids! So this is a disadvantage with respect to free drugs.

**Figure. Penetration of therapeutics/nanomedicine (5FU-CUR-PIP-HSA-NPs) in MDA-MB-231 cancer cells in 3D tumor spheroids.** In vitro. In vitro uptake of free drugs combination and 5FU-CUR-PIP-HSA-NPs with different concentrations and incubation times in 3D tumor spheroids. The green fluorescence represents CUR. Scale bars = 200 μm.

It is not stated in the paper that NPs could not penetrate tight spheroids. As shown in the figure above, compared to the free drug combination group, the NPs achieved a homogenous radial distribution in tumor spheroids. It was observed that free drugs were mostly confined to the periphery of the tumor spheroids. On the other hand, deeper penetration was observed for the NPs formulation into the tumor spheroids (even into their necrotic core) which is in accordance with the enhanced penetration of the NPs formulations than free drugs reported in the literature [4, 61-66]. The drug carrier helped the penetration into the tumor spheroids enhancing the depth of NPs penetration compared to the free drugs. Proper size, zeta potential, and drug combination of the prepared drug delivery system in albumin NPs could be responsible for the facilitated effective deep penetration into the solid tumors in good agreement with previous studies [2, 67-69].

A phrase in the conclusion part is also amended as follows to avoid ambiguities:

“Relative to the cell monolayers, higher concentrations of drugs were needed to be employed for both free drugs combination and NPs formulation to induce cytotoxicity in spheroids due to the limited transport of the drugs/NPs into the tight structure of the microtissues and their intrinsic drug resistance” (Section 4. Conclusion, page 27)

9) The statement about HTS with plates and models is not justified. The authors present a screen done in low adhesion 96 well plates. What is new about this? What is different from any other HTS assays in plates (e.g. 96 or even higher 384 well plates)?

This question is addressed in the detailed explanation provided for question #2 as well.

In the ultra-low attachment plates, agar/agarose and poly(2-hydroxyethyl methacrylate) (pHEMA) are mainly used to coat the plates for spheroid culture [2]. The transferring step need for subsequent bioassays and the short storage time of the coated plates have been considered the main disadvantage of using agar/agarose in the liquid overlay technique [3, 18], while long-term culture may be needed for spheroids to form and become dense [4, 7]. In addition, when using flat non adherent surfaces, several irregular cellular aggregates can be formed limiting the high-throughput screening applications [18]. Besides, pHEMA is relatively expensive, and preparing the pHEMA-coated plates is laborious [18]. Moreover, compared with homemade coated plates, commercially available low-adhesion 96-well plates are relatively expensive [27].

Due to long cultivation time, cumbersome culturing technique, formation of unequal-size spheroid and failure to produce rigid homogenous spheroids, a high throughput screening on spheroids is limited in several current platforms. Spheroid cultures of homogenous sizes and growth characteristic are important factors that greatly affect the precise quantification of biological or biochemical endpoints in drug screening. [3]. Moreover, harvesting the spheroids from the culture platform such as microfluidic devices and hanging drops poses a challenge for high throughput screening. Spheroids are often lost during medium exchange [34].

To address these challenges, Utilizing a homemade set-up, we developed a scaffold-free 3D spheroid culture platform in standard 96-well flat plates in a cost-effective novel approach for rapid generation of homogenous single tight 3D tumor spheroids in each well in a U-shape design. In order to prepare this platform we used PDMS casting and surface modification with PVA. PDMS, as a biocompatible cost-effective polymer with several excellent material properties for biomedical applications provides a chemically inert surface with low interfacial free energy, good thermal stability, and optical transparency. The surface properties of PDMS are also relatively easy to modify [28]. Compared with other spheroid models the proposed platform in this study presents several advantages. Such round-shaped spheroids produced in this model can benefit mathematical modeling too.

A large number of homogenous highly reproducible spheroids can be produced in the prepared platform, compatible with the high-throughput screening assays. Using this 3D culture platform, spheroids can be arrayed and utilized to screen chemical or RNAi/CRISPR libraries in combination with a variety of assay readouts such as size, viability, and imaging without needed transferring steps. The effect of chemical compounds like therapeutics on a variety of parameters such as viability, MTS size, reporter genes, or fluorescent dye intensity can also be assessed [29]. In fact, screening techniques currently applied to monolayer cultures in multiwell plate formats, such as various assays for cytotoxicity, proliferation, drug binding, apoptosis, and ATP level, can be adapted to spheroids and could potentially be automated into a high throughput approach.

By integrating other elements (e.g. co-culture), our high throughput compatible platform has the potential to be employed as an intermediate step to enhance the translational predictive value of *in vitro* drug evaluation studies and provide a reduction in both financial and time costs during later stages of the drug development timeline, allowing the early detection of ineffective agents, thus reducing the risk of drug withdrawal from the market [30]. The procedure is potent to be extended to primary patient samples (organoid) for drug screening too.

The disadvantages of other employed methods by researchers are highlighted and the advantages of utilization of our platform for rapid generation of reproducible homogenous tumor spheroids with high compactness for high throughput screening assays and readouts are mentioned throughout the paper and the manuscript is justified accordingly. The below statements are added to the manuscript as well:

“To address these, herein, we established a preclinical scaffold-free 3D spheroid culture model in the standard 96-well flat plates by employing a homemade set-up. The novel aspect of our 3D platform includes the generation of a large quantity of uniform tight single spheroids in a cost and time-effective approach for drug screening with no necessary transfer step. Using this new in situ high throughput compatible 3D model system alongside 2D culture platforms, we investigated the efficacy of the formulated drug delivery system (5FU-CUR-PIP-HSA-NPs) to triple negative MDA-MB-231 breast cancer cells. The study provides proof-of-concept results for employing the 3D culture system to test the biological activities of the drug delivery system.” (Section 1. Introduction, page 5)

“To address these challenges, a particular treated concave-based bed in a 96-well plate (high throughput compatible) with a U-shape design was established in this study for rapid one spheroid formation in each well. PDMS, as a biocompatible cost-effective polymer with several excellent material properties for biomedical applications provides a chemically inert surface with low interfacial free energy, good thermal stability, and optical transparency. The surface properties of PDMS are also relatively easy to modify (54).” (Section 3.5. Formation and characterization of multicellular tumor spheroids, page 18)

“Spheroid cultures of uniform sizes and growth characteristics are critical factors that considerably affect the accurate quantification of biological or biochemical endpoints in drug/nanomedicine screening (31). Compared with the conventional techniques like hanging drop, this platform can rapidly generate reproducible homogenous large tumor microtissues with high compactness paving the way for a variety of high throughput screening assays and readouts with no transferring steps.” (Section 3.5. Formation and characterization of multicellular tumor spheroids, page 19)

10) Figure 9 is just an estimate schematic, to show proliferative, quiescent and necrotic zones, but specific dyes should be used to show the different phenotypes in a spheroid.

large spheroids (starting from about 500 μm in diameter) are characterized by an external proliferating zone, an internal quiescent zone caused by the limited distribution of oxygen, nutrients, and metabolites, and a necrotic core resembling the cellular heterogeneity of solid in vivo tumors [29, 34, 70]. As the spheroids produced in our study exceed 500 μm in diameter, we inferred that the representative three concentric zones of proliferative, quiescent, and necrotic regions must be present in them. The darkest region of a spheroid imaged in brightfield is reported to be mainly composed of quiescent/dead cells [70]. To further verify this we also performed a live/dead assay on the produced tumor spheroid with FDI/PI staining. The red fluorescence highlighted an inner core composed of only dead, non-viable cells that corresponding to the darkest region in the brightfield image.

**Figure.** Fluorescent image of live/dead assay on the tumor spheroid using FDA (green) and PI (red)). Scale bar = 200 μm

The cellular layers of a spheroid reflect the in vivo TME with actively proliferating tumor cells on the periphery and distant inner cells remaining in a quiescent state or undergoing cell death due to necrosis and apoptosis [17].

The live/dead assay figure is added to the manuscript in the combined figure (Fig 4B) and the below statement is added to the manuscript:

“The existence of the necrotic core was substantiated by the live/dead staining of a tumor spheroid (Fig 4B).” (Section 3.5. Formation and characterization of multicellular tumor spheroids, page 20)

Reviewer #3: The work has good design and contains valuable information on “Rapid Generation of Homogenous Tumor Spheroid Microtissues in a Scaffold-Free Platform for High-Throughput Screening of a Novel Combination Nanomedicine”. I am sure that the paper will give further insights into new studies related to tumor spheroids for the screening of nanomedicines. However, I suggested the paper acceptance after major corrections.

Thank you very much for your time and constructive comments.

1. Introduction part of the manuscript is too long. Please re-write and reduce the size of the introduction to almost half.

The introduction part of the manuscript is rewritten and the length of it is significantly reduced.

2. The methodology part is unclear. This seems like the author skipped many important information.

The material and method section (section 2) was tried to be as detailed and clear as possible. Some additional information is added to the methodology part, especially regarding 2D and 3D cell cultures for bioassays. The section 2. Materials and methods is complemented with more information and proper references are added to the methodology part to enhance its clarity.

The study’s minimal underlying data set is also provided in a Supporting Information file named “S1 File”.

3. Why did the author use PVA coating instead of using low attachment plates for generating 2D and 3D spheroids?

For 2D cell culture, standard flat-bottom 96-well plates with no treatment were used.

Employing the liquid overlay technique principles, a promising scaffold-free platform was developed in standard 96-well flat plates in a cost-effective novel approach for rapid generation of homogenous tight 3D tumor spheroid microtissues and anticancer therapeutic/nanomedicine screening. The technological aspects and superiority of the developed platform over other commonly used spheroid culture techniques were discussed in the manuscript and summarized in S1 Table. A detailed explanation is provided below to justify using our prepared 3D platform in this study:

One frequently used method for spheroid fabrication is the liquid overlay method [1]. In the ultra-low attachment plates, generally, the main substrates used to coat the plate are agar/agarose or poly(2-hydroxyethyl methacrylate), by adding 50 μL of solution in each well of 96-well plates, at concentrations of 10-15 mg/mL and 5mg/mL, respectively [2]. Agar/agarose [3-10] and poly(2-hydroxyethyl methacrylate) [11, 12] coated 96-well plates and commercially available low adhesion 96-well plates [13-16] have been extensively used for spheroid culture and drug screening.

However, the variability of the size and/or spherical morphology of the spheroids, low efficiency, limited visualization, lack of reproducibility, and difficulty in long-term culture have been counted as disadvantages for most employed ultra-low attachment plates [17-22]. The biomaterials used for the non-adherent surface culture so far, could have interactions with the drug or nano-carriers, thus affecting the accuracy of the results acquired from spheroid models as well [23].

The short storage time of the coated plates has been considered the main disadvantage of using agar/agarose in the liquid overlay technique [18]. Water evaporation and consequently the coating disruption can occur in such covered plates during various days and result in impairing the reproducible production of the spheroids. Furthermore, long-term culture may be needed for spheroids to form and become dense for biological activity tests [4, 6, 7, 24]. transferring step for subsequent drug treatment, imaging, and analysis is majorly required in such coated plates [3] increasing the risk of disaggregation and/or loss of spheroid structure.

Likewise, the utilization of poly(2-hydroxyethyl methacrylate), as a non-adherent material for the production of spheroids through the liquid overlay technique, is relatively expensive and laborious [18]. Moreover, compared with in-house coated plates, commercially available low-adhesion 96-well plates are relatively expensive [25-27].

In this study, PDMS, as a biocompatible cost-effective polymer with several excellent material properties for biomedical applications provides a chemically inert surface with low interfacial free energy, good thermal stability, and optical transparency. The surface properties of PDMS are also relatively easy to modify [28]. In our work, by using PDMS casting in 96-well plates, an in-house U-shape design to the bottom of the culture plate wells was established (concave bottom) and modified with PVA to prevent cell adhesion to the surface and facilitate single and well-defined round shape spheroid formation per wells. A large number of homogenous highly reproducible spheroids can be produced in the cost-effective prepared platform, compatible with the high-throughput screening assays. This method is user-friendly and can be ready-to-use in many labs.

when using flat non-adherent surfaces with a thin layer of non-adherent biomaterial, several irregular cellular aggregates with non-spherical shapes have been obtained by the liquid overlay technique [18]. These cellular aggregates do not meet the requirements to be used in high-throughput screening, which hinders the translation of these technologies to the industry [18]. The round-shaped single spheroids cultured in our model can benefit mathematical modeling too [18]. With the calculated diameter in the round-shaped tumor spheroids produced in our platform, a good estimate can be performed on the volume ($V=\frac{1}{6}\left( \pi D^{3} \right)$) and the number of cells within spheroids and drug/nanodrug distribution across the spheroids can be mathematically modeled.

In addition, due to the difficulty to obtain homogenous spheroid cultures, a high throughput screening on spheroids is limited [3]. Scaffold-free systems in 96-well plates format are currently the most amenable to high-throughput screening, due to their compatibility with conventional liquid handling and robotic equipment [29]. In this regard, large number of homogenous highly reproducible spheroids were produced in the prepared platform, compatible with the high-throughput screening assays. Using this 3D culture platform, spheroids can be arrayed and utilized to screen chemical or RNAi/CRISPR libraries in combination with a variety of assay readouts such as size, viability, and imaging without transferring steps. The effect of the chemical compounds like therapeutics on a variety of parameters such as viability, MTS size, reporter genes, or fluorescent dye intensity can also be assessed [29]. Accordingly, the use of spheroids can be extended for more practical disease modeling and high-throughput screening.

The explanations and points are added to the manuscript to justify it and the aims of using the prepared 3D platform in this work are discussed accordingly. The added statements to the manuscript are as follows:

“The aim of this study was to develop a homemade preclinical 3D culture model in the standard 96-well plates in a cost and time-effective novel approach for the rapid generation of homogenous compact tumor spheroids for disease modeling, and anticancer therapeutic/nanomedicine screening.” (Abstract, Page 2)

“To address these, herein, we established a preclinical scaffold-free 3D spheroid culture model in the standard 96-well flat plates by employing a homemade set-up. The novel aspect of our 3D platform includes the generation of a large quantity of uniform tight single spheroids in a cost and time-effective approach for drug screening with no necessary transfer step. Using this new in situ high throughput compatible 3D model system alongside 2D culture platforms, we investigated the efficacy of the formulated drug delivery system (5FU-CUR-PIP-HSA-NPs) to triple negative MDA-MB-231 breast cancer cells. The study provides proof-of-concept results for employing the 3D culture system to test the biological activities of the drug delivery system.” (Section 1. Introduction, page 5)

“In the ultra-low attachment plates, agar/agarose and poly(2-hydroxyethyl methacrylate) (pHEMA) are mainly used to coat the plates for spheroid culture (4). The transferring step need for subsequent bioassays and the short storage time of the coated plates have been considered the main disadvantage of using agar/agarose in the liquid overlay technique (30, 31), while long-term culture may be needed for spheroids to form and become dense (51, 52). In addition, when using flat non adherent surfaces, several irregular cellular aggregates can be formed limiting the high-throughput screening applications (30). Besides, pHEMA is relatively expensive, and preparing the pHEMA-coated plates is laborious (30). Moreover, compared with homemade coated plates, commercially available low-adhesion 96-well plates are relatively expensive (53).” (Section 3.5. Formation and characterization of multicellular tumor spheroids, page 18)

“To address these challenges, a particular treated concave-based bed in a 96-well plate (high throughput compatible) with a U-shape design was established in this study for rapid one spheroid formation in each well. PDMS, as a biocompatible cost-effective polymer with several excellent material properties for biomedical applications provides a chemically inert surface with low interfacial free energy, good thermal stability, and optical transparency. The surface properties of PDMS are also relatively easy to modify (54).” (Section 3.5. Formation and characterization of multicellular tumor spheroids, page 18)

“Spheroid cultures of uniform sizes and growth characteristics are critical factors that considerably affect the accurate quantification of biological or biochemical endpoints in drug/nanomedicine screening (31).” (Section 3.5. Formation and characterization of multicellular tumor spheroids, page 19)

“Compared with the conventional techniques like hanging drop, this platform can rapidly generate reproducible homogenous large tumor microtissues with high compactness paving the way for a variety of high throughput screening assays and readouts with no transferring steps.” (Section 3.5. Formation and characterization of multicellular tumor spheroids, page 19)

“Such round-shaped spheroids produced in this model can benefit mathematical modeling too (30).” (Section 3.5. Formation and characterization of multicellular tumor spheroids, page 20)

“As a practical 3D model, a novel scalable scaffold-free production of controllable tumor spheroids platform was established in this study by utilizing a homemade set-up for disease modeling and anticancer drug/nanomedicine screening” (Section 4. Conclusion, page 26)

4. There were no clear information about 3D spheroid formation in the same methodology part.

The spheroid culture procedure is complemented with more information and references as follows:

“Rapid scaffold-free spheroid culture of MDA-MB-231 cells was performed in a cost-effective novel approach by employing the liquid overlay technique principles (30). A treated concave-based bed was provided for cells in 96-well flat-bottom plates to form homogenous tumor spheroids (Figs 1A and 1B). Initial optimization tests were implemented to define the optimum values affecting the process (eg. PDMS volume, coating substrate, cell density, culture time). To this end, a pre-polymer of PDMS and its curing agent was prepared in a ratio of 10:1 w/w, stirred manually, and degassed in a vacuum desiccator. 50 μl of the mixture was poured into each well of the 96-well plate and allowed to settle and cast. The plate was placed in the incubator and the PDMS layer was cured at 37˚C for 5 h. Then, 50 μl of PVA solution (1% w/v in water) was poured into each well and incubated under a cell culture hood at room temperature for 24 h. The surface of the PDMS-cured 96-well plate was treated with PVA to prevent cell adhesion to the surface and facilitate tumor spheroid formation. The next day, the dried treated wells were washed with PBS and the plates were sterilized using UV for 30 min.

**Figure 1. 3D platform for spheroid culture.** (A) Spheroid culture process onto the non-adherent concave bed in a 96-well plate. (B) peeled off PDMS layer from a well.

In order to produce large spheroids, high cell numbers have been used per well in 96-well plates (31-34). The size of the spheroids can be manipulated by varying seeded cell numbers and culture periods (32). MDA-MB-231 cells were reported to have a diameter and volume of 14.3 µm and 1530 µm3, respectively (35). Taking the ideal round shape and high tightness for tumor spheroids into account, the approximate number of cells needed for the generation of tumor spheroids with a desired size range was calculated using Eq. (4).

$$V=\frac{1}{6}\left( \pi d^{3} \right) \left( 4 \right)$$

Where D and V are the diameter and volume of the tumor spheroids, respectively. Performing some pretests under these assumptions, a cell number range of 20,000-40,000 was selected to obtain spheroids with a size range of 400-500 µm for different biological activity tests. Employing a high cell density of 106 cells/ml, cells were seeded onto each non-adherent round-bottomed well and were allowed to settle into the recesses. The medium then was added to each well to reach a volume of 200 μl. 50% of the culture medium was exchanged every other day during the tissue growth. Tumor spheroid microtissues were grown for 72 h to bear a compact structure before the biological activity tests. Spheroid formation was monitored over time using an Optika microscope (Italy) with 4X and 10X magnifications.” (Section 2.4. 2D cell culture and 3D spheroid formation, pages 9-11).

5. Did author use EGF and FGF growth factors in the growth media of tumor spheroids??

No growth factors were used in the growth media for tumor spheroid culture.

6. The number of cells used for spheroids formation is too high for 96 well plates. The author should provide the proper justification with citations for using that much high number of cells.

In order to generate large spheroids, high cell numbers have been used in 96-well plates in several studies [3, 5, 71-73]. Varying seeded cell numbers and culture periods were reported to enable researchers to control the size of the produced spheroids [71]. 300-500 μm was reported to be the optimum diameter size for tumor spheroids to practically mimic the *in vivo* conditions in terms of hypoxia and proliferation gradients [2]. The round-shaped single spheroids cultured in our model can benefit mathematical modeling [18].

Considering the sphere shape for the cells and the high tightness of the tumor spheroids, the number of cells needed for spheroid generation in the size range of 400-500 µm was estimated. MDA-MB-231 cells were reported to have a diameter and volume of 14.3 µm and 1530 µm^3^, respectively [74]. Taking an ideal sphere morphology from the geometric mean of 2 orthogonal diameters for generated tumor tissues into account, the approximate number of cells needed for producing tumor spheroids with the desired size was calculated by equation below.

$V=\frac{1}{6}\left( \pi d^{3} \right)$

Where d and V are the diameter and volume of the tumor spheroid, respectively. Under these assumptions, to obtain spheroids with the size range of 400-500 µm, approximately, the cell number range of 21891-42756 would be needed. Performing some pretests, a cell number range of 20000-40000 was selected for spheroid generation in different biological activity tests.

The justification for using high cell numbers for spheroid culture is added to the manuscript as follows:

“In order to produce large spheroids, high cell numbers have been used per well in 96-well plates (31-34). The size of the spheroids can be manipulated by varying seeded cell numbers and culture periods (32). MDA-MB-231 cells were reported to have a diameter and volume of 14.3 µm and 1530 µm3, respectively (35). Taking the ideal round shape and high tightness for tumor spheroids into account, the approximate number of cells needed for the generation of tumor spheroids with a desired size range was calculated using Eq. (4).

$$V=\frac{1}{6}\left( \pi d^{3} \right) \left( 4 \right)$$

Where D and V are the diameter and volume of the tumor spheroids, respectively. Performing some pretests under these assumptions, a cell number range of 20,000-40,000 was selected to obtain spheroids with a size range of 400-500 µm for different biological activity tests” (Section 2.4. 2D cell culture and 3D spheroid formation, page 10)

7. Cell density used per well for scratch wound healing assay is also very high as author used 24 wells plate. Please provide references considering during the experiment performed.

To provide the desired confluency, high cell numbers of cancer cells have been used per well in 24-well plates for the scratch wound healing assay [54, 75-77]. Based on “Surface Areas and Guide for Recommended Medium Volumes for Corning® Cell Culture Vessels” (<https://www.corning.com/catalog/cls/documents/application-notes/CLS-AN-209.pdf>) the average cell yield for a 24-well plate is 1.9 × 10^5^ in each well. In order to obtain 70% monolayer confluency for scratch wound healing assay, we made a proportion and performed some pretests; then we seeded 140,000 cells/well.

The justification for using high cell numbers in the wound healing assay is added to the manuscript as follows:

“In order to provide the desired confluency, high cell numbers of MDA-MB-231 cancer cells can be used per well in 24-well plates for the scratch wound healing assay (37, 38).” (Section 2.9. Scratch wound healing assay, page 13)

8. In many places the author cited the mechanism such as drug induces tumor cell apoptosis via various apoptosis signaling pathways such as NF-kB, PI3K/Akt, and STAT3. This is totally irrelevant in the whole manuscript as the author did not do anything related to mechanism throughout his study.

The sentences mentioning signaling pathways such as NF-kB, PI3K/Akt, and STAT3 are deleted from the manuscript as you recommended.

9. Figure 2B: I would like to see the complete crop image of Particle Size Distribution Graph mentioning the size of particles.

Complete results of the DLS analysis of the NPs including the size of particles and PDI are provided in the figure below. The analysis was replicated 3 times and intensity size and PDI were reported as mean ± standard deviation (SD).

**Figure.** Complete results of the DLS analysis including the size of particles and PDI of 5FU-CUR-PIP-HSA-NPs.

10. I do not find the Zeta Potential Graph of particle. I would recommend please including the Zeta potential graph as well as PDI values.

The PDI value is included in the DLS results. For determining the surface charge of the NPs we utilized ZETA-check (Particle Metrix, Germany). As shown in the figure below the device has a monitor on itself that shows the magnitude of the surface charge while it does not create graphs. We recorded the data shown on the screen of the device, calculated the mean and standard deviation, and reported them in the manuscript.

**Figure.** ZETA-check (Particle Metrix) instrument used in this work for determining the surface charge of the 5FU-CUR-PIP-HSA-NPs.

11. There are no scale bars in figure 10, figure 11, and figure 13. Please provide the scale bars for all these images.

The scale bars are added to the figures as you recommended.

**Figure 5. Penetration of therapeutics/nanomedicine (5FU-CUR-PIP-HSA-NPs) in MDA-MB-231 cancer cells in 2D and 3D conditions.** In vitro uptake of (A) free drugs combination and (B) 5FU-CUR-PIP-HSA-NPs in 2D monolayer cancer cells at the CUR/5FU/PIP concentration of 50/26.8/7.4 μg/ml after 4 h. Scale bars = 50 μm. In vitro uptake of (C) free drugs combination and (D) 5FU-CUR-PIP-HSA-NPs with different concentrations and incubation times in 3D tumor spheroids. The green fluorescence represents CUR. Scale bars = 200 μm.

**Figure.** Migration inhibition of metastatic MDA-MB-231 cells by wound healing assay. After the scratch creation, cells were treated with 5FU-CUR-PIP-HSA-NPs. After 24 h, the wound’s gap became completely covered in the control group, meanwhile, the migration of metastatic cells was suspended using a low concentration of the NPs. Scale bars = 50 μm.

12. I suggested language checking of the manuscript before publication because some mistakes are looked in the manuscript.

The manuscript is double-checked and errors were corrected. The changes made to the manuscript are highlighted in the “Revised Manuscript with Track Changes” file.

13. Please include some important references during revising the manuscript suggested below:

https://doi.org/10.1016/j.fct.2019.01.018
https://doi.org/10.1016/j.mtchem.2021.100618
<https://doi.org/10.1016/j.msec.2019.110285>

Thank you for suggesting the relevant important references. The papers are utilized and cited in the manuscript as follows:

“Phytochemicals with biological effects against cancer cells can be used in combination with existing chemotherapeutics to enhance tumor therapy (6)” (Section 1. Introduction, page 3)

“Dissimilar to the reported data (69, 70), even at high concentrations, no destruction in the structure or reduction in size was observed in the tumor spheroids after the treatments.” (Section 3.7. Anticancer activity in 2D and 3D culture platforms, page 25)

While revising your submission, please upload your figure files to the Preflight Analysis and Conversion Engine (PACE) digital diagnostic tool, <https://pacev2.apexcovantage.com/>. PACE helps ensure that figures meet PLOS requirements. To use PACE, you must first register as a user. Registration is free. Then, login and navigate to the UPLOAD tab, where you will find detailed instructions on how to use the tool. If you encounter any issues or have any questions when using PACE, please email PLOS at [figures@plos.org](mailto:figures@plos.org). Please note that Supporting Information files do not need this step.

The figures are comprehensively edited and combined. The figures are also edited and standardized using the PACE service of PLOS ONE as well.

**References**

[1] Lu H, Stenzel MH. Multicellular tumor spheroids (MCTS) as a 3D in vitro evaluation tool of nanoparticles. Small. 2018;14:1702858.

[2] Pinto B, Henriques AC, Silva P, Bousbaa H. Three-dimensional spheroids as in vitro preclinical models for cancer research. Pharmaceutics. 2020;12:1186.

[3] Ho WY, Yeap SK, Ho CL, Rahim RA, Alitheen NB. Development of multicellular tumor spheroid (MCTS) culture from breast cancer cell and a high throughput screening method using the MTT assay. 2012.

[4] Sarisozen C, Dhokai S, Tsikudo EG, Luther E, Rachman IM, Torchilin VP. Nanomedicine based curcumin and doxorubicin combination treatment of glioblastoma with scFv-targeted micelles: In vitro evaluation on 2D and 3D tumor models. European journal of pharmaceutics and biopharmaceutics. 2016;108:54-67.

[5] Metzger W, Sossong D, Bächle A, Pütz N, Wennemuth G, Pohlemann T, et al. The liquid overlay technique is the key to formation of co-culture spheroids consisting of primary osteoblasts, fibroblasts and endothelial cells. Cytotherapy. 2011;13:1000-12.

[6] Sarisozen C, Abouzeid AH, Torchilin VP. The effect of co-delivery of paclitaxel and curcumin by transferrin-targeted PEG-PE-based mixed micelles on resistant ovarian cancer in 3-D spheroids and in vivo tumors. European Journal of Pharmaceutics and Biopharmaceutics. 2014;88:539-50.

[7] Bugno J, Hsu H-J, Pearson RM, Noh H, Hong S. Size and surface charge of engineered poly (amidoamine) dendrimers modulate tumor accumulation and penetration: A model study using multicellular tumor spheroids. Molecular pharmaceutics. 2016;13:2155-63.

[8] Nguyen HN, Ha PT, Sao Nguyen A, Nguyen DT, Do HD, Thi QN, et al. Curcumin as fluorescent probe for directly monitoring in vitro uptake of curcumin combined paclitaxel loaded PLA-TPGS nanoparticles. Advances in Natural Sciences: Nanoscience and Nanotechnology. 2016;7:025001.

[9] Mussi SV, Sawant R, Perche F, Oliveira MC, Azevedo RB, Ferreira LA, et al. Novel nanostructured lipid carrier co-loaded with doxorubicin and docosahexaenoic acid demonstrates enhanced in vitro activity and overcomes drug resistance in MCF-7/Adr cells. Pharmaceutical research. 2014;31:1882-92.

[10] Huang K, Ma H, Liu J, Huo S, Kumar A, Wei T, et al. Size-dependent localization and penetration of ultrasmall gold nanoparticles in cancer cells, multicellular spheroids, and tumors in vivo. ACS nano. 2012;6:4483-93.

[11] Ivascu A, Kubbies M. Rapid generation of single-tumor spheroids for high-throughput cell function and toxicity analysis. Journal of biomolecular screening. 2006;11:922-32.

[12] Ivascu A, Kubbies M. Diversity of cell-mediated adhesions in breast cancer spheroids. International journal of oncology. 2007;31:1403-13.

[13] Alzeeb G, Arzur D, Trichet V, Talagas M, Corcos L, Jossic-Corcos L. Gastric cancer cell death analyzed by live cell imaging of spheroids. Scientific reports. 2022;12:1-13.

[14] Mikhail AS, Eetezadi S, Ekdawi SN, Stewart J, Allen C. Image-based analysis of the size-and time-dependent penetration of polymeric micelles in multicellular tumor spheroids and tumor xenografts. International journal of pharmaceutics. 2014;464:168-77.

[15] Mikhail AS, Eetezadi S, Allen C. Multicellular tumor spheroids for evaluation of cytotoxicity and tumor growth inhibitory effects of nanomedicines in vitro: a comparison of docetaxel-loaded block copolymer micelles and Taxotere®. PloS one. 2013;8:e62630.

[16] Zhao J, Lu H, Wong S, Lu M, Xiao P, Stenzel MH. Influence of nanoparticle shapes on cellular uptake of paclitaxel loaded nanoparticles in 2D and 3D cancer models. Polymer Chemistry. 2017;8:3317-26.

[17] Khawar IA, Ghosh T, Park JK, Kuh H-J. Tumor spheroid-based microtumor models for preclinical evaluation of anticancer nanomedicines. Journal of Pharmaceutical Investigation. 2021;51:541-53.

[18] Costa EC, de Melo‐Diogo D, Moreira AF, Carvalho MP, Correia IJ. Spheroids formation on non‐adhesive surfaces by liquid overlay technique: Considerations and practical approaches. Biotechnology journal. 2018;13:1700417.

[19] Katt ME, Placone AL, Wong AD, Xu ZS, Searson PC. In vitro tumor models: advantages, disadvantages, variables, and selecting the right platform. Frontiers in bioengineering and biotechnology. 2016;4:12.

[20] Benien P, Swami A. 3D tumor models: history, advances and future perspectives. Future oncology. 2014;10:1311-27.

[21] Jubelin C, Muñoz-Garcia J, Griscom L, Cochonneau D, Ollivier E, Heymann M-F, et al. Three-dimensional in vitro culture models in oncology research. Cell & Bioscience. 2022;12:1-28.

[22] Hu Q, Gao X, Kang T, Feng X, Jiang D, Tu Y, et al. CGKRK-modified nanoparticles for dual-targeting drug delivery to tumor cells and angiogenic blood vessels. Biomaterials. 2013;34:9496-508.

[23] Huang B-W, Gao J-Q. Application of 3D cultured multicellular spheroid tumor models in tumor-targeted drug delivery system research. Journal of controlled release. 2018;270:246-59.

[24] Mei L, Fu L, Shi K, Zhang Q, Liu Y, Tang J, et al. Increased tumor targeted delivery using a multistage liposome system functionalized with RGD, TAT and cleavable PEG. International journal of pharmaceutics. 2014;468:26-38.

[25] Breslin S, O’Driscoll L. Three-dimensional cell culture: the missing link in drug discovery. Drug discovery today. 2013;18:240-9.

[26] Zanoni M, Pignatta S, Arienti C, Bonafè M, Tesei A. Anticancer drug discovery using multicellular tumor spheroid models. Expert opinion on drug discovery. 2019;14:289-301.

[27] Li M, Song X, Jin S, Ye K. 3D tumor model biofabrication. Bio-Design and Manufacturing. 2021;4:526-40.

[28] Miranda I, Souza A, Sousa P, Ribeiro J, Castanheira EM, Lima R, et al. Properties and applications of PDMS for biomedical engineering: A review. Journal of Functional Biomaterials. 2021;13:2.

[29] Calpe B, Kovacs WJ. High-throughput screening in multicellular spheroids for target discovery in the tumor microenvironment. Expert Opinion on Drug Discovery. 2020;15:955-67.

[30] Barbosa MA, Xavier CP, Pereira RF, Petrikaitė V, Vasconcelos MH. 3D Cell Culture Models as Recapitulators of the Tumor Microenvironment for the Screening of Anti-Cancer Drugs. Cancers. 2021;14:190.

[31] Pampaloni F, Reynaud EG, Stelzer EH. The third dimension bridges the gap between cell culture and live tissue. Nature reviews Molecular cell biology. 2007;8:839-45.

[32] Nunes AS, Barros AS, Costa EC, Moreira AF, Correia IJ. 3D tumor spheroids as in vitro models to mimic in vivo human solid tumors resistance to therapeutic drugs. Biotechnology and bioengineering. 2019;116:206-26.

[33] Hamilton G, Rath B. Applicability of tumor spheroids for in vitro chemosensitivity assays. Expert Opinion on Drug Metabolism & Toxicology. 2019;15:15-23.

[34] Mehta G, Hsiao AY, Ingram M, Luker GD, Takayama S. Opportunities and challenges for use of tumor spheroids as models to test drug delivery and efficacy. Journal of controlled release. 2012;164:192-204.

[35] Henke E, Nandigama R, Ergün S. Extracellular matrix in the tumor microenvironment and its impact on cancer therapy. Frontiers in molecular biosciences. 2020;6:160.

[36] Herrmann D, Conway JR, Vennin C, Magenau A, Hughes WE, Morton JP, et al. Three-dimensional cancer models mimic cell–matrix interactions in the tumour microenvironment. Carcinogenesis. 2014;35:1671-9.

[37] Machado Brandão-Costa R, Helal-Neto E, Maia Vieira A, Barcellos-de-Souza P, Morgado-Diaz J, Barja-Fidalgo C. Extracellular matrix derived from high metastatic human breast cancer triggers epithelial-mesenchymal transition in epithelial breast cancer cells through αvβ3 integrin. International journal of molecular sciences. 2020;21:2995.

[38] Daryasari MP, Akhgar MR, Mamashli F, Bigdeli B, Khoobi M. Chitosan-folate coated mesoporous silica nanoparticles as a smart and pH-sensitive system for curcumin delivery. Rsc Advances. 2016;6:105578-88.

[39] Kim T-H, Mount CW, Gombotz WR, Pun SH. The delivery of doxorubicin to 3-D multicellular spheroids and tumors in a murine xenograft model using tumor-penetrating triblock polymeric micelles. Biomaterials. 2010;31:7386-97.

[40] Hiremath CG, Kariduraganavar MY, Hiremath MB. Synergistic delivery of 5-fluorouracil and curcumin using human serum albumin-coated iron oxide nanoparticles by folic acid targeting. Progress in biomaterials. 2018;7:297-306.

[41] Xiao Y, Liu Y, Yang S, Zhang B, Wang T, Jiang D, et al. Sorafenib and gadolinium co-loaded liposomes for drug delivery and MRI-guided HCC treatment. Colloids and Surfaces B: Biointerfaces. 2016;141:83-92.

[42] Monajati M, Tavakoli S, Abolmaali SS, Yousefi G, Tamaddon A. Effect of PEGylation on assembly morphology and cellular uptake of poly ethyleneimine-cholesterol conjugates for delivery of sorafenib tosylate in hepatocellular carcinoma. BioImpacts: BI. 2018;8:241.

[43] Wang X, Zhen X, Wang J, Zhang J, Wu W, Jiang X. Doxorubicin delivery to 3D multicellular spheroids and tumors based on boronic acid-rich chitosan nanoparticles. Biomaterials. 2013;34:4667-79.

[44] Rawal S, Patel MM. Threatening cancer with nanoparticle aided combination oncotherapy. Journal of controlled release. 2019;301:76-109.

[45] Gao Y, Tang M, Leung E, Svirskis D, Shelling A, Wu Z. Dual or multiple drug loaded nanoparticles to target breast cancer stem cells. RSC Advances. 2020;10:19089-105.

[46] Anitha A, Sreeranganathan M, Chennazhi KP, Lakshmanan V-K, Jayakumar R. In vitro combinatorial anticancer effects of 5-fluorouracil and curcumin loaded N, O-carboxymethyl chitosan nanoparticles toward colon cancer and in vivo pharmacokinetic studies. European Journal of Pharmaceutics and Biopharmaceutics. 2014;88:238-51.

[47] Gong C, Deng S, Wu Q, Xiang M, Wei X, Li L, et al. Improving antiangiogenesis and anti-tumor activity of curcumin by biodegradable polymeric micelles. Biomaterials. 2013;34:1413-32.

[48] Pachauri M, Gupta ED, Ghosh PC. Piperine loaded PEG-PLGA nanoparticles: Preparation, characterization and targeted delivery for adjuvant breast cancer chemotherapy. Journal of Drug Delivery Science and Technology. 2015;29:269-82.

[49] Maia FR, Reis RL, Oliveira JM. Nanoparticles and Microfluidic Devices in Cancer Research. Biomaterials-and Microfluidics-Based Tissue Engineered 3D Models: Springer; 2020. p. 161-71.

[50] Boix-Montesinos P, Soriano-Teruel PM, Armiñán A, Orzáez M, Vicent MJ. The past, present, and future of breast cancer models for nanomedicine development. Advanced drug delivery reviews. 2021;173:306-30.

[51] Michy T, Massias T, Bernard C, Vanwonterghem L, Henry M, Guidetti M, et al. Verteporfin-loaded lipid nanoparticles improve ovarian cancer photodynamic therapy in vitro and in vivo. Cancers. 2019;11:1760.

[52] Li H-J, Du J-Z, Liu J, Du X-J, Shen S, Zhu Y-H, et al. Smart superstructures with ultrahigh pH-sensitivity for targeting acidic tumor microenvironment: instantaneous size switching and improved tumor penetration. ACS nano. 2016;10:6753-61.

[53] Pershina AG, Brikunova OY, Demin AM, Abakumov MA, Vaneev AN, Naumenko VA, et al. Variation in tumor pH affects pH-triggered delivery of peptide-modified magnetic nanoparticles. Nanomedicine: Nanotechnology, Biology and Medicine. 2021;32:102317.

[54] Ma L, Lin X, Li C, Xu Z, Chan C-Y, Tse M-K, et al. A cancer cell-selective and low-toxic bifunctional heterodinuclear Pt (IV)–Ru (II) anticancer prodrug. Inorganic Chemistry. 2018;57:2917-24.

[55] Sadreddini S, Safaralizadeh R, Baradaran B, Aghebati-Maleki L, Hosseinpour-Feizi MA, Shanehbandi D, et al. Chitosan nanoparticles as a dual drug/siRNA delivery system for treatment of colorectal cancer. Immunology letters. 2017;181:79-86.

[56] Sarkar A, Ghosh S, Chowdhury S, Pandey B, Sil PC. Targeted delivery of quercetin loaded mesoporous silica nanoparticles to the breast cancer cells. Biochimica et Biophysica Acta (BBA)-General Subjects. 2016;1860:2065-75.

[57] Ghosh S, Dutta S, Sarkar A, Kundu M, Sil PC. Targeted delivery of curcumin in breast cancer cells via hyaluronic acid modified mesoporous silica nanoparticle to enhance anticancer efficiency. Colloids and Surfaces B: Biointerfaces. 2021;197:111404.

[58] Paudel KR, Wadhwa R, Tew XN, Lau NJX, Madheswaran T, Panneerselvam J, et al. Rutin loaded liquid crystalline nanoparticles inhibit non-small cell lung cancer proliferation and migration in vitro. Life sciences. 2021;276:119436.

[59] Mahalunkar S, Yadav AS, Gorain M, Pawar V, Braathen R, Weiss S, et al. Functional design of pH-responsive folate-targeted polymer-coated gold nanoparticles for drug delivery and in vivo therapy in breast cancer. International journal of nanomedicine. 2019;14:8285.

[60] Lu M, Wang S, Khine YY, Hong Y, Zheng J, Lu H, et al. Dual drug delivery system of RAPTA-C and paclitaxel based on fructose coated nanoparticles for metastatic cancer treatment. Biochemical and Biophysical Research Communications. 2022.

[61] Lu H, Utama RH, Kitiyotsawat U, Babiuch K, Jiang Y, Stenzel MH. Enhanced transcellular penetration and drug delivery by crosslinked polymeric micelles into pancreatic multicellular tumor spheroids. Biomaterials science. 2015;3:1085-95.

[62] Singh SP, Sharma M, Gupta PK. Cytotoxicity of curcumin silica nanoparticle complexes conjugated with hyaluronic acid on colon cancer cells. International journal of biological macromolecules. 2015;74:162-70.

[63] Taresco V, Abelha TF, Cavanagh RJ, Vasey CE, Anane‐Adjei AB, Pearce AK, et al. Functionalized Block Co‐Polymer Pro‐Drug Nanoparticles with Anti‐Cancer Efficacy in 3D Spheroids and in an Orthotopic Triple Negative Breast Cancer Model. Advanced Therapeutics. 2021;4:2000103.

[64] Singh SP, Sharma M, Gupta PK. Evaluation of phototoxic effects of curcumin loaded in organically modified silica nanoparticles in tumor spheroids of oral cancer cells. Bionanoscience. 2015;5:10-21.

[65] Perche F, Patel NR, Torchilin VP. Accumulation and toxicity of antibody-targeted doxorubicin-loaded PEG–PE micelles in ovarian cancer cell spheroid model. Journal of controlled release. 2012;164:95-102.

[66] Dilnawaz F, Sahoo SK. Enhanced accumulation of curcumin and temozolomide loaded magnetic nanoparticles executes profound cytotoxic effect in glioblastoma spheroid model. European Journal of Pharmaceutics and Biopharmaceutics. 2013;85:452-62.

[67] Priwitaningrum DL, Blondé J-BG, Sridhar A, van Baarlen J, Hennink WE, Storm G, et al. Tumor stroma-containing 3D spheroid arrays: A tool to study nanoparticle penetration. Journal of controlled release. 2016;244:257-68.

[68] Wilhelm C, Billotey C, Roger J, Pons J, Bacri J-C, Gazeau F. Intracellular uptake of anionic superparamagnetic nanoparticles as a function of their surface coating. Biomaterials. 2003;24:1001-11.

[69] Wenzel C, Riefke B, Gründemann S, Krebs A, Christian S, Prinz F, et al. 3D high-content screening for the identification of compounds that target cells in dormant tumor spheroid regions. Experimental cell research. 2014;323:131-43.

[70] Zanoni M, Piccinini F, Arienti C, Zamagni A, Santi S, Polico R, et al. 3D tumor spheroid models for in vitro therapeutic screening: a systematic approach to enhance the biological relevance of data obtained. Scientific reports. 2016;6:1-11.

[71] Huang Y-C, Chan C-C, Lin W-T, Chiu H-Y, Tsai R-Y, Tsai T-H, et al. Scalable production of controllable dermal papilla spheroids on PVA surfaces and the effects of spheroid size on hair follicle regeneration. Biomaterials. 2013;34:442-51.

[72] Kim M, Yun H-W, Park DY, Choi BH, Min B-H. Three-dimensional spheroid culture increases exosome secretion from mesenchymal stem cells. Tissue engineering and regenerative medicine. 2018;15:427-36.

[73] Monjaret F, Fernandes M, Duchemin-Pelletier E, Argento A, Degot S, Young J. Fully automated one-step production of functional 3D tumor spheroids for high-content screening. Journal of Laboratory Automation. 2016;21:268-80.

[74] Wagner BA, Venkataraman S, Buettner GR. The rate of oxygen utilization by cells. Free Radical Biology and Medicine. 2011;51:700-12.

[75] Wu X-X, Yue GG-L, Dong J-R, Lam CW-K, Wong C-K, Qiu M-H, et al. Actein inhibits the proliferation and adhesion of human breast cancer cells and suppresses migration in vivo. Frontiers in pharmacology. 2018;9:1466.

[76] Abolghasemi R, Ebrahimi-Barough S, Bahrami N, Aid J. Atorvastatin inhibits viability and migration of MCF7 breast cancer cells. Asian Pacific Journal of Cancer Prevention: APJCP. 2022;23:867.

[77] Zhou Y-C, Liu J-Y, Li J, Zhang J, Xu Y-Q, Zhang H-W, et al. Ionizing radiation promotes migration and invasion of cancer cells through transforming growth factor-beta–mediated epithelial–mesenchymal transition. International Journal of Radiation Oncology* Biology* Physics. 2011;81:1530-7.
